# What Can We Learn from Dissecting Tortricid Females About the Efficacy of Mating Disruption Programs?

**DOI:** 10.3390/insects16030248

**Published:** 2025-02-28

**Authors:** Alan Lee Knight, Michele Preti, Esteban Basoalto

**Affiliations:** 1 Instar Biologicals, Yakima, WA 98908, USA; 2 Independent Integrated Pest Management Consultant and Researcher, 48018 Faenza, Italy; 3 Facultad de Ciencias Agrarias, Instituto de Producción y Sanidad Vegetal, Universidad Austral de Chile, Valdivia 5110566, Chile

**Keywords:** virgin and mated females, delay of mating, moth immigration, female behavior, pheromone autodetection

## Abstract

Female mating success for the tortricids codling moth (CM), *Cydia pomonella*, Oriental fruit moth (OFM), *Grapholita molesta*, European grape vine moth (EGVM), *Lobesia botrana*, and five leafroller (LR) species under various mating disruption (MD) programs was reviewed at a time when new dual sex lures can provide alternative tools to assess female mating. Previous reliance on passive assessments such as tethering and virgin female-baited traps with laboratory moths are at odds with active trapping methods of wild moths. Additive factors such as delayed mating, adjustments in female behaviors, and greater levels of natural control may or may not contribute to the apparent success of MD. Current MD programs are not based solely on research, as economics and commercialization require some compromise. The complete sex pheromone blend is not always used. A delay in mating has been reported from the field with one study and suggested that reductions in fecundity would likely be minimal. There is no evidence that MD works better with low population densities. MD is an established technology, but the new dual sex lures are showing that the density of mated females is rather high. Efforts to improve the efficacy of MD are ongoing with a small cadre of researchers.

## Simple Summary

Sex pheromones, naturally released by female moths to attract males, have been synthesized and released at high levels to achieve mating disruption. Unfortunately, it has been more difficult to measure actual female mating disruption than communication disruption of males to synthetic lures placed in traps. Here we summarize the literature on several key tortricids that are important pests of tree fruit and grapes and for which over 1,000,000 hectares are treated with mating disruption products. Two types of assessments (stationary and active) have been used to sample female mating, and they differ widely in their estimates of mating success. We present limitations associated with both types of measures. A review of the literature suggests female moth behaviors have not been well considered in defining the success of mating disruption. The recent identification of dual sex lures for these tortricids may allow another and simpler technique to be used to estimate female mating success. These lures can be used to compare different types (hand-applied dispensers, sprayables, and aerosols) and combinations of these mating disruption techniques in order to develop more effective management programs.

## 1. Introduction

The focus of this review is the literature on the mating status of key female tortricid pests (Lepidoptera: Tortricidae) attacking tree fruit and vineyards treated with a mating disruption (MD) technique. Adult tortricids are small moths whose larvae feed internally or externally on fruits and can cause very high levels of crop damage [1]. Key tortricids can have a narrow or broad host range, feeding on wild plants and unmanaged hosts surrounding commercial plantings. The expansion of peri-urban agriculture can be the source of unmanaged insect populations that are an increasing threat to pest management and require a cooperative spirit [2]. Tortricids are key pests in fruit crops such as apples or grapes, requiring an integrated management program of best practices [3,4]. The adoption of MD programs for these pests has often allowed growers to use less insecticide while maintaining the high commodity standards required by the consumer [3,5].

Mating disruption is a general term to describe a sex pheromone-based practice used to reduce the proportion of moth females that can mate, are delayed in mating, or somehow have their behaviors impacted, resulting in reduced oviposition. The success of MD technology is pragmatically supported by its adoption on more than one million hectares [6]. Specifically, nearly 700,000 hectares of orchards and vineyards are treated with MD to manage just three tortricid pests: codling moth (CM), *Cydia pomonella* L.; Oriental fruit moth (OFM), *Grapholita molesta* Busck; and European grape vine moth (EGVM), *Lobesia botrana* Denis & Shiffermüller. Another 75,000 hectares are treated with MD for other tortricids, including numerous leafroller (LR) species.

Interestingly, the complete literature on characterizing female mating levels of these species under MD is not extensive and mostly includes bait and light traps or placing laboratory-reared virgin females tethered to foliage or placed in traps to attract wild males. However, over the last 10 years, several dual sex attractant blends (i.e., effective for both males and females) have been developed for female tortricids, and they can be used in standard monitoring traps to assess levels of female mating within MD-treated tree fruit and grapes. The use of synthetic lures to monitor female tortricids under MD provides new and more accessible data. The efficacies of the different MD programs embedded with supplemental insecticide sprays for each pest are not fully understood. Reductions in male moth catch in sex pheromone-baited traps are insufficient to describe MD. We suggest that MD programs cannot be optimized until we understand the full impact of MD on female moths. The current testing of these dual sex lures varies widely among the tortricid pests selected for this review. In addition, questions are not fully answered concerning whether these new data obtained on female mating are biased. Finally, the negative impact of female moth immigration into MD-treated areas is recognized but has not been quantified or well managed.

This review compares the available data on female mating under MD from diverse studies in tree fruits and grapes with recent data from the use of dual sex lures, attractive to both male and female moths. The reviewed data were collected with either stationary or active assessments, and, in general, these two classes of sampling produced different results and perhaps tell separate stories about MD. The possible explanations for these differences are discussed. Finally, the available data on the importance and potential of MD in delaying mating and impacting female tortricids’ responses to sex pheromones (autodetection) are summarized.

## 2. Background

Mating disruption is an established component of management programs in tree fruits and grapes. Dispenser technologies, pest population density, surrounding unmanaged hosts, moth dispersal, and orchard characteristics such as size, shape, slope, aspect, and canopy uniformity all impact MD [7]. Pest density is a major factor shaping the success of MD. This relationship is often misunderstood, and practitioners stress that MD works better at lower densities [6,8]. Crop injury is impacted more by pest population density than by female mating success. Yet, outside of studies conducted in small cages, few have compared levels of female mating as a function of density under MD [9,10]. Organizing CM field data based on the numbers of females caught in 180 traps baited with pear ester (ethyl (*E*,*Z*)-2,4-decadienoate) found there was little relationship of female density over a 10-fold range with their proportional mating success [11].

Mechanisms associated with the use of MD include sensory adaptation, competition, and camouflage [12]. The role of these mechanisms likely varies in importance under different versions of MD, which include variable densities of hand-applied dispensers, coverage with microencapsulated sprayables, and low-point source density emitters such as aerosol units [6]. The mechanisms of MD have been biased towards understanding the success of males searching for females and not as much with female behaviors when exposed to MD practices [13].

The development of effective dispensers for MD has been based on careful behavioral research, technological innovations, and trial and error. The amount of sex pheromone released per hectare over time was developed from the initial work with the cabbage looper, *Trichoplusia ni* (Hübner) (*Lepidoptera*: *Noctuidae*) [14]. This led to extensive testing of dispensers to establish the minimum effective pheromone concentration to disrupt male orientation [15]. Three subsequent decades of pheromone dispenser development allowed the commercialization and adoption of MD technologies ranging from hand-applied dispensers to sprayables and aerosols. Studies to optimize these technologies have tested variable rates and point source densities, and technological improvements and economies of scale eventually pushed each technology to its current labeled rates [16,17,18,19]. It is not clear whether the current use rates of MD are optimal to disrupt female mating. This review presents how the research measuring the disruption of female mating played a necessary yet insufficient role in this process.

An important assumption in MD development has been that a complete sex pheromone blend should be more effective than a partial blend [20]. All the tortricids included in this review have a suite of compounds identified from sex gland extracts, but reduced synthetic blends of one to four components are used in commercial lures and dispensers. For example, the sex pheromone blend for female CM was identified as nine components, but only one component was found to be necessary for maximum male captures in traps, namely codlemone, (*E*,*E*)-8,10-Dodecadien-1-ol [21]. Similarly, commercial products for EGVM use a single major component, (*E*,*Z*)-7,9-dodecadienyl acetate, despite the identification of several biologically active secondary compounds [8]. Early studies with OFM found that a two-component blend was less effective than three components [15,22], while the four-component blend later identified is not practically used [23]. Nearly all the MD studies with LR have been comparisons of two- to four-component blends of 14-carbon chain-length acetates, alcohols, and aldehydes with varying (*Z*,*E*) isomeric ratios [24]. Differences in the ‘female potency’ among these key tortricids, perhaps based on female size, has been noted [25]. Larger tortricids such as oblique banded leafroller (OBLR), *Choristoneura rosaceana* Harris, and CM are more difficult to disrupt than the species with smaller size adults [6]. Developing effective MD has also tested pheromone antagonists and para-pheromones [26,27].

## 3. Monitoring Female Tortricids Under MD

Many reviews of tortricid MD have been written since Cardé and Minks [28] shortsightedly predicted that MD for CM was not likely to be successful, leading to a recent, more positive view [29]. Improved monitoring is often listed in these reviews as a key factor needed to better evaluate MD [6,7,30,31,32]. Yet, most studies on tortricid MD have not reported the mating status of females, likely because there have been few useful methods to gather these data in field conditions.

Interest in developing new female-targeted lures is correlated with the increasing labor cost of visually sampling pests and the need to seasonally track pest populations. Sampling life stages other than adults early in the season before crop injury occurs is always used in MD orchards [8,33]. For example, counting eggs is practical for many LR species because they deposit masses that are easy to sample when populations are large [34]. In contrast, the individual eggs laid by CM and OFM females are not easily found. Pear ester increased CM oviposition and affected egg distribution on branches, but egg densities were too low to develop a practical tool [35,36]. Anecdotally, one pear consultant in California developed an artificial fruit injury method to sample CM eggs based on his observation that eggs on pears were often placed near fruits injured by bird pecks [37]. Counting EGVM eggs in grape clusters early in the season has been used to assess the effect of MD, but developing an artificial surface and effective lure from host plant volatiles to monitor eggs laid by the attracted females was not possible [38,39,40]. Broken shoot tips and rolled leaves are common sampling methods to assess potential damage early- and mid-season for OFM and LR species, respectively [41]. Corrugated tree bands have been used to assess the density of overwintering CM larvae and to track yearly pest population changes under MD [42].

Developing more effective monitoring of MD is a key factor in developing integrated programs, including new thresholds and spray timings based on female sampling [43,44,45]. Sex pheromone lures for male tortricids are probably the least expensive and easiest tool to assess MD success [46]. Only one study has developed a tool to measure mating of both sexes [47]. Thus, a male catch in traps serves as a place card for the number of mated females. Unfortunately, sex pheromone-baited traps can be disrupted by MD, which limits their ability to assess pest population densities.

There are two separate uses for female lures. The first is to track the seasonal flight of the sex most directly related to crop injury. The second and the focus of this review is to develop an effective lure to monitor female mating under different types of MD. Lures developed with plant and microbial-derived volatile organic compounds can perhaps serve these roles more effectively than sex pheromone lures under MD [48]. Trap placement relative to fruit can impact the number of females trapped [49,50], while proximity to sex pheromone dispensers does not seem to be as important [50,51]. Smart traps are being developed to allow remote monitoring due to labor constraints for some growers [52]. Separation of the sexes in these traps may be possible by measuring sexual differences in size and wing vibration, but likely not their mating status [53,54].

The importance of developing standardized tools to assess the effectiveness and registration of MD tactics has been recognized [55]. Assessment errors with male lures can be caused by the variability among sex pheromone lures and their decay over time and the use of higher, non-female-equivalent emission rates with the lures [56,57]. Sex pheromone-baited traps can register false negative assessments of MD [43]. Checking the mating status of females placed or trapped within MD treatments would be a more direct assessment. The tools that can be used to assess female mating in MD plots can be characterized as one of two types: stationary and active assessments.

Stationary assessments use virgin female-baited traps and tethered virgin females (VF) placed in the canopy as a ‘sentinel’ [58]. The proportion of traps catching > 1 male or the mean catch by a cohort of traps can be used to measure the impact of MD as well as the proportional mating status of the tethered females when compared to untreated plots. These two methods allow an assessment of MD without the impact imposed by wild-mated females immigrating into the treated blocks. The stationary assessments have some limitations, which are described hereafter. Researchers typically place tethered females and traps in optimal positions, though they rarely report their position in relation to MD dispensers. Both methods utilize virgin females and thus require an insect colony. Laboratory-reared moths are very short-lived when transferred from the laboratory to hot summer weather in the field. Predation of tethered moths can be very high in unsprayed crops from vespids and spiders. Tethering the smaller species, such as OFM and EGVM, is more difficult than tethering CM and LR and is less frequently reported. The biggest problem with interpreting these methods’ results is likely restraining the virgin female from moving throughout the canopy. The positioning of calling females in the canopy has been reported for at least one species [59]. But their movement and repositioning within the crop are not easily studied [60]. Also, the limited exposure of female moths tethered for 24 h to the volatiles released by the MD dispensers may minimize the full behavioral impact from habituation/adaptation [61]. For example, laboratory studies have found that female calling and flight behavior of OFM and OBLR can be impacted for at least 24 h following sex pheromone exposure [62]. The pre-exposure of conspecific pupae and 2–3 d old adults under the laboratory rearing conditions may already impact the tethered females. Studies have not characterized the behaviors of tethered females.

Active assessments are made with wild females that actively fly into traps. Traps can include clear oil-coated passive interception traps, UV light traps, or traps with attractive semiochemical lures, including both non-selective baits and moderately selective host plant and/or microbial-produced volatiles. The clear interception trap is a useful research tool, catching both sexes [63]. Mark–recapture studies with only female moths with a known initial mated proportion demonstrated that this trap was not biased for mated or virgin females [64]. Baiting these traps with pear ester lures can increase female catch up to 10-fold over the standard delta trap [65]. One unique direct sampling method used for CM involved vacuuming off all the moths resting on trees, but they did not dissect the females [66]. The limitations of the active assessments are listed below. Sugary fermenting baits and UV light traps were the tools used to time sprays before sex pheromones were identified, and with OFM, their use continued in MD-treated orchards [67,68,69]. Sugary fermenting baits degrade over time and are very non-selective, requiring frequent and laborious handling [70,71]. They catch mostly mated females and spray timing had to be adjusted based on the physiological age of the females [67,72,73]. It is not clear whether these catches are biased or reflect the resident population’s mating status—[74] vs. [75]. The AJAR bait trap, which places the bait inside a delta trap and can catch moths on a sticky liner, was more effective and easier to use than standard pails, but this design is not available commercially [71,76,77]. No study has compared proportions of mated vs. virgin females using mark–recapture in pest-free plots. One limitation of oil-coated interception traps is that they catch much fewer moths per trap and are messy and short-lived [63].

Light traps have been shown to be more effective than bait traps and catch physiologically younger CM females [78,79,80]. Tortricid photoreceptors respond primarily to two peaks in light radiation (UV, 340–420 nm, and green, 460–540 nm), but UV lights tend to be the most effective [81,82,83,84,85]. Light traps are expensive, and it can be difficult to separate out the target moth from the assemblage of several orders of non-targets, which was previously impractical in large plantings distant from a power source. Light traps are no longer widely used to monitor tortricids and are more commonly deployed as ‘bug zappers’. The recent development of inexpensive solar-powered LED lights has made the supplemental use of lights in IPM more feasible [86]. The range of attraction of standard light traps can be large [87]; however, traps with a single UV-A LED are attractive over a shorter range and catch fewer non-targets [84,85].

The variable levels of female immigration into MD trials can strongly impact active assessment methods for female mating status. Many MD studies have been conducted in plots ≤2 ha and are often surrounded by untreated host crops. Assessing the mating of wild female moths in small plots is a problem not necessarily alleviated by monitoring borders and centers of plots separately [88,89].

## 4. Female Removal Techniques

Another use of sex pheromones has been for mass trapping and/or attracting and killing [90,91]. Models demonstrate that adequate crop protection with tactics only removing male moths can be problematic [92,93]. Successful use of attracticides for male tortricids has involved applying thousands of point sources per hectare, often with specialized equipment, and can require multiple applications [94]. This approach was considered more viable for small and irregular-shaped orchards in Europe than for the large rectangular plantings in western North America. Commercialization of several products ultimately failed after a decade of testing. Formulations requiring females to touch a pesticide residue have not been successfully developed; however, early trials with insecticide-treated netting were promising but were later discontinued. Mass trapping with female attractants has been moderately effective for CM, alone or in combination with MD, but faces registration constraints and is likely not competitive with MD and the continued reliance on cheap insecticides [95,96,97].

## 5. Areawide and Spatial Exclusion

Mating disruption used over large contiguous multi-grower areas for these tortricid pests has been organized and has been very successful [3,5,29,98,99,100,101,102,103,104]. Fundamentally expanding the boundaries of a management program so that the potential immigration of mated females is negligible is a key characteristic of successful programs [7]. Secondly, their success comes from the organization and overall improved management associated with these well-funded government-in-partnership-with-grower programs [5].

The identification and removal of problem pest sites within the large management unit was an important factor that allowed the neighbors to be successful [105]. None of these areawide programs included an assessment of female moth mating.

A second method of restricting the pest from attacking the crop is the use of exclusion netting [106,107]. Tight netting on the tree canopy apparently restricts sexual communication and female mating of CM [106]. Only one study assessed the female mating status of both wild and released sterile CM under netting [107]. Canopy structure was found to be a key factor affecting mating. Interestingly, netted cages have also been used to assess MD success [108].

## 6. Sterile Moth Release Programs

Sterile release programs with tortricid pests have been conducted for CM in Canada [109]. In addition, several pilot programs have been evaluated for the light brown apple moth, *Epiphyas postvittana* Walker (Lepidoptera: Tortricidae), CM in New Zealand, and EGVM in Chile [110]. Some programs have combined the use of sterile moth releases and MD [109,111]. Program successes have largely been tracked by measuring the overflooding ratio of sterile:wild males caught in sex pheromone-baited traps [112]. However, these data may not reflect the success of the sterile insect technique [113]. The mating status of the sterile females and their ability to mate is checked in the laboratory [111]. Virgin female-baited traps and tethered females have been used experimentally to characterize the competitiveness of sterile vs. wild moths [114,115]. But no operational effort has been made by the program to assess the proportion of sterile:wild mating, especially in the pockets of remaining infestations. The collection of wild and sterile female CM in live traps could be used to ascertain the proportion of sterile:wild matings by examining the fertility of eggs.

## 7. Effective Volatiles

Volatiles that are attractive to female tortricids have been investigated for 40 years [116]. The sophistication of gas chromatography analysis and the development of electroantennography technology allowed researchers to survey the activity of an array of host volatiles [117,118]. This research increased dramatically with the adoption of MD to develop more effective monitoring lures in a sex pheromone-saturated environment [119]. In some cases, these volatiles have led to new action thresholds and improved spray timing [43,44,45]. A series of studies in China have evaluated host volatiles from peach and pear to improve monitoring of the female OFM, but they have not reported the females’ mating status [120,121].

Interestingly, laboratory-based assays of female behaviors to selected volatiles have not led to any commercial attractants despite some early claims of attraction [122,123,124,125,126]. Acetic acid is the one volatile that is consistent in all of the blends that are attractive, but it has very little attractiveness when used alone [127]. The hypothesis that unmated females fly to food sources while mated females fly to host volatiles does not seem to fit the extensive data for tortricids [128]. The development of a non-sex pheromone quaternary dual sex lure for CM, comprising linalool oxide, pear ester, DMNT ((*E*)-4,8-Dimethyl-1,3,7-nonatriene), and acetic acid, occurred through personal synergy and created a supernatural flower–fruit–leaf and fermentation blend not found in nature [50,129,130]. This unexpected complexity suggests that molecular efforts to identify new attractants by characterizing one odorant receptor at a time are not likely to be successful in the near term [8,119].

## 8. New Dual Sex Lures

Over the last 10 years, different dual sex lures have been developed for each of these species. Interestingly, the development of pear ester-based lures, including pear ester, acetic acid, and DMNT, never reported the mating status of females [119]. Combining pear ester with DMNT, linalool oxide, and acetic acid into an effective quaternary non-pheromone-based lure for CM in 2018 initially did not report the mating status of females [129,130,131]. Similarly, articles published on the development of OFM lures combining the sex pheromone of OFM and CM plus terpinyl acetate and acetic acid only reported female mating status in one [71] out of five studies [76,132,133,134]. The initial development of dual attractants for LR and EGVM using 2-phenylethanol or phenylacetonitrile in combination with acetic acid also focused only on the sex ratio of moths [135,136,137,138,139,140,141,142]. To date, the mating status of female moths caught with these lures has been reported from two studies with OBLR and EGVM, respectively [143,144]. This omission in a decade-long research of dual sex lures was likely because pest managers had clearly expressed that they were not going to sex, let alone dissect females. Secondly, the mating status of female moths under MD apparently was no longer considered to be important data needed to assess the success of MD based on these omissions. One exception was that the level of multiple-mated female CM was found to be a useful indicator of MD’s effectiveness [96,145,146,147]. The level of female mating (virgin, single-mated, or multiple-mated) is usually assessed by dissecting the female abdomen and observing the bursa copulatrix using a stereomicroscope. The shape and size of the female spermatheca allow us to determine the female mating status, and the number of spermatophores contained allows us to determine the number of matings [146].

Since 2017, our studies with non-sex pheromone lures have always reported the mating status of female moths. We collect these data to evaluate the effect of immigration and the relative mating status of females under different MD programs. A solar-powered UV-A LED added to delta traps baited with these lures is a significant improvement in collecting female moths [84,85]. The mating status of CM, OFM, EGVM, and OBLR has been reported using this bi-modal trapping approach from orchards treated with several MD programs from one or more countries, including the USA, Uruguay, Chile, and Spain [84,85,144].

## 9. Review of Literature

Using primarily Google Scholar, a careful selection of 60 papers reporting the mating status of female moths of the abovementioned tortricid species under 176 MD programs was obtained. Specifically, the literature related to CM (Table A1), OFM (Table A2), EGVM (Table A3), and five LR species (Table A4) was grouped by pest species and organized in different trials according to the experiment characteristics (trials #1–82 for CM, trials #83–118 for OFM, trials #119–129 for EGVM, and trials #130–176 for LR species). The focus of these studies varied among evaluations of attractive blends, emission rates, and point source densities. Both active and stationary assessment techniques were utilized; however, the types of techniques employed varied widely among species. Data are provided when available for the percentage of mated females in a control and under MD. The percentage reduction in this ratio is also calculated (Table A1, Table A2, Table A3 and Table A4, Appendix A). Several types of analyses were conducted comparing assessment methods for each pest, and for CM and LR, the effect of tested blends was also compared.

All statistical analyses were performed with R software (v. 4.4.2). Data derived from the cited references were separated by pest species and organized according to their type as percentage of reduction in female mating in different MD treatments compared to their controls (extrapolated from the published results of virgin and mated females), percentage of reduction in multiple-mated females (considering only references with results specification between single and multiple matings), and percentage of reduction in trap catching at least one male in different MD treatments compared to their controls (extrapolated from the published results of total male catches). Similar studies with the same type of data collected using comparable materials and methods were used as independent replicates to test the effect of either the MD treatment or the assessment method. The data were analyzed according to their distribution. Data normality was tested using Shapiro–Wilk’s test. Linear models (ANOVA test) were used with normal data, while data found to be close to a normal distribution were transformed using the square root function (sqrt (x + 0.5)). Data that were not normally distributed were analyzed using the non-parametric Kruskal–Wallis test by rank. Akaike’s information criteria (AIC) and residuals were both used to select the fitted models for each analysis. A multiple comparison post-hoc test was performed on the fitted models, and Tukey’s HSD test (*p* < 0.05) was used to discriminate significant differences among treatments. Treatments with an insufficient number of replicates (<3) were not included in the statistical analyses. All data presented are the mean values ± standard error of the mean (SE).

### 9.1. Codling Moth

There were 30 publications with 82 studies of CM that reported female mating (Table A1). Twelve studies did not have a comparison of both treated and untreated orchards. Studies either used the two stationary assessments [39] and/or several types of active assessments [43], including passive interception traps and several types of pear ester-based lures (Table 1). No bait pails were reported, and only two light trap studies were found, and these did not compare the effect of MD vs. no treatment. The most common MD tactic was the use of 800–1000 dispensers ha^−1^. Dispensers were either loaded with one or three components of the CM sex pheromone (PH) or just the major pheromone component (i.e., codlemone) with pear ester (PH/PE). The data from fiber tapes, sprayables (microencapsulated, MEC), and aerosols were collected but were too limited to summarize as individual groups.

Nine compounds were identified in gland extracts of female CM [21], but previously, flight tunnel assays found that the blend was not more attractive to males than the major component, codlemone, alone [148]. A second flight tunnel study with males, however, suggested a five-compound blend was more attractive than the single major component [149]. This blend included both dodecanol and tetradecanol, the two components included in the Isomate dispensers [150]. Other studies have considered the use of codlemone acetate (*E*,*E*)-8,10-dodecadien-1-yl acetate alone or in combination with codlemone dispensers [151,152].

The stationary vs. active assessment methods provided quite different evaluations of MD technologies for CM (Table 1). The two stationary methods found a significantly greater reduction in mating than with either active method, i.e., passive interception traps and traps with pear ester-based lures. The two stationary assessments were not different from each other, but the tethered female method was somewhat more sensitive to disruption. This could be affected by the typical use of more than one virgin female per trap compared with a single moth being tethered.

The PH/PE dispensers using the tethered female assessment were significantly more effective than PH dispensers: mean reduction in mating = 92.5 vs. 83.2, W = 4.0482, *p* = 0.0442. While the mean reduction in traps catching at least one male was similarly higher, 78.5 vs. 69.5, with the virgin female-baited trap technique under PH/PE vs. PH treatments, this difference was not significant, F_1,15_ = 0.27, *p* = 0.6125.

The performance of the interception traps was variable among the seven trials, likely due to significant immigration levels from unmanaged blocks in two studies [11,61]. Interception traps were found previously to provide an unbiased measure of the proportion of mated females in a population [64]. Thus, the data from these traps may be the most accurate measure of the range of female CM mating under MD.

Light traps were used in older studies to monitor CM and not to assess females in any of the early MD trials. The addition of a single LED to delta traps has significantly increased male and female catches of CM [84]. However, it has not affected the proportion of female mating.

Four variants of pear ester-based lures have been used to capture female CM, pear ester alone, in combination with sex pheromone alone and with acetic acid, and the four-component lure comprised pear ester, DMNT, linalool oxide, and acetic acid. Pear ester, when used alone as a lure, provided a significantly lower reduction in the percentage of mated females under MD (5.3 ± 1.8) than when used with sex pheromone PH/PE ‘Combo’ lure (13.4 ± 2.6) or using the four-component lure (14.3 ± 3.2), which were not different from each other (F_2,21_ = 3.83, *p* = 0.0381).

The occurrence of multiple matings by female CM was recorded in 12 studies, all using pear ester-based lures. The percent reduction in multiple-mated female CM was much greater under MD (mean = 65.1 ± 6.9) than for all females mated in the same studies (mean = 9.6 ± 2.1), with a significant difference (F_1,22_ = 50.97, *p* < 0.0001). There was no difference (F_1,10_ = 0.42, *p* = 0.5331) in the percentage decrease in multiple mating by CM females between PH (mean = 47.9 ± 6.6) and PH/PE (mean = 51.0 ± 8.5) dispensers.

The large number of CM studies allowed us to compare the percentage decline in female mating using dispensers applied at the same density but loaded with either PH or PH/PE using several assessment methods. Two studies compared Isomate PH dispensers with Cidetrak PH/PE using tethered females, and the latter dispenser reduced mating by an additional 6% [153,154]. The proportion of VF-baited traps catching males was reduced by 10, 25, and 20% more in three studies with CIDETRAK^®^ PH/PE vs. either Isomate or CIDETRAK^®^ PH dispensers [155,156]. The mean reduction in males catch using VF-baited traps was reduced 41% more with CIDETRAK^®^ PH/PE vs. CIDETRAK^®^ PH dispensers and was reduced by 20% using PI traps [61]. CIDETRAK^®^ MESO PH/PE dispensers applied at 80 units ha^−1^ reduced mating 9 and 32% more compared with CIDETRAK^®^ MESO PH dispensers [147]. One exception was found in trials with MEC formulations, where the PH MEC outperformed the PH/PE MEC applications by 10% [157]. Yet, a second MEC study found the PH/PE formulation slightly outperforming the PH by 5% in walnuts [145]. The low volume application (46 L ha^−1^) of a MEC formulation reduced the proportion of VF-baited traps catching males by 33% compared with the standard air blast spray at 926 L ha^−1^ due to enhanced deposition of capsules [158]. Two studies compared the reductions in the mating of tethered females (Isomate vs. NoMate) and the proportion of VF traps catching males (chopped fiber) between PH dispensers with one or three components [159,160]. The two blends performed similarly in both studies. There were two other avenues of research with CM MD that were not explored over an extended period, although they showed some early promise. The use of codlemone acetate (anti-pheromone) achieved 100% disruption of VF traps [151], and a decade later, an equilibrium isomeric blend of 8,10-dodecadienol increased disruption by 18–24% over codlemone alone using VF traps [161]. These studies were not included in Table 1 due to their limited testing and absence in the current marketplace.

The PE MEC formulation used alone for MD was only reported in one study [157]. The PE MEC sprays are used more frequently by walnut and apple growers to enhance insecticide-based control [162,163]. Four applications of PE MEC timed for MD had a minimal impact (3–12%) on reducing the proportion of mated females and did not improve the PH MEC when used together [157]. The addition of PE MEC to PH MEC significantly decreased the proportion of multiple-mated CM females in walnuts in one out of two years and reduced nut injury [145]. Other studies have found that PE MEC significantly reduced levels of injury when used in combination with PH or PH/PE dispensers [61,162].

Whether the mostly low to moderate reductions in the percentage of mated CM females reported with MD using pear ester-based lures is accurate has not been fully evaluated. Only one study reported female mating in programs with commercial dispensers used at a higher than standard rate, 2000 dispensers ha^−1^, and this was assessed with virgin female-baited traps [164]. Unfortunately, with this passive assessment, both 1000 and 2000 dispensers ha^−1^ reduced male catch by 100%. Only one study used both interception and PE-baited traps together [11]. The two measures of female mating were similar, but lure-baited traps estimated a smaller disruption effect. A summary of more recent data using the quaternary dual sex-based lure found a three-fold greater reduction in percent mating than previously estimated with the PE lure [50]. This suggests that the four-component lure may provide a similar estimate to interception traps of the impact of CM MD on female mating, but direct comparative studies are still needed. Also, studies addressing whether the mating status of female CM caught with this lure is biased have not yet been completed.

Among tortricid species, CM has been categorized as a difficult pest to disrupt with sex pheromones [6]. The disruption of CM male catch in sex pheromone-baited traps is clearly impacted by dispenser density or the total amount of pheromone released [16]. There are currently at least four companies that have hand-applied dispensers, aerosols, and sprayables for CM MD in the USA market. Future studies are needed to characterize the effectiveness of the many types and possible combinations of CM MD that this array of products allows. A particular focus will be on growers with significant CM management issues to create complex MD programs deploying more than one tactic. The effectiveness of these new programs can be ascertained via reductions in female mating, not male catch in traps. A standard protocol with releases of sterilized virgin CM adults obtained from the Okanagan–Kootenay Sterile Moth Program in Osoyoos, British Columbia, can be used to measure reductions in female mating under this diversity of choices.

### 9.2. Oriental Fruit Moth

Bucket traps with terpinyl acetate and brown sugar bait have been used for a century to monitor OFM populations [165], and their use was extended into the 21st century in some MD-treated orchards [68,69,166]. Bait traps typically catch more than 60% female OFM, but their measure of female mating under MD has not always been useful [74]. Some studies using bait traps to assess OFM MD have not even reported female catch or their mating status [68,166]. Early evaluations of MD for OFM with bait traps were usually in small blocks surrounded by other non-disrupted hosts [167]. The movement of OFM populations from stone to pome fruit orchards can be significant and requires areawide management to be effective [100]. Unfortunately, female mating status in the middle of these large areawide studies with multiple hosts was not reported.

The influence of dispenser emission rate and point source densities are key factors influencing MD success. The different methods used to assess female mating prevent a clear evaluation of these factors. Similar to the data summarized for CM, the percent reductions in the mating of tethered OFM females in four studies and the catch of males in virgin female-baited traps in eight studies have been high under MD, i.e., 75–100%. However, this large reduction has not always been useful in predicting MD’s effectiveness [168].

Studies where baits have been a useful tool to measure OFM female mating under MD have been ignored [75]. Two more recent studies have recorded <30% mated females in bait traps under MD [71,77]. Dry combination lures of terpinyl acetate and acetic acid also caught low numbers of mated female OFM [132]. Unfortunately, only one of these studies compared mating levels of populations in MD-treated vs. untreated orchards [75]. Interestingly, the 76% reduction in mating assessed with baits was comparable to stationary assessment methods (Table 1).

Commercial development of a combinational dual lure comprised sex pheromone together with food attractants created a much more effective tool to replace sex pheromone lures for OFM under MD [133,134]. Interestingly, so far, its use has not measured any significant reductions in OFM female mating under MD [85]. The addition of a UV LED to traps significantly increased moth catches, but with a similar percentage of mated females [84,85]. Trials monitored with this OFM combinational dual lure have included two hand-applied and aerosol dispensers. The data were consistent and suggested that MD was only moderately effective in disrupting female mating in these orchards. However, many of these blocks were small and surrounded by non-disrupted hosts. A wider adoption of such a combinational dual lure to assess the effectiveness of a diversity of management program for OFM can be likely useful for growers and pest control advisers. The ability to measure the mating success of OFM field populations could allow refinement in detailing the required point source densities and atmospheric concentrations with different commercial products. Interestingly, the combination of the OFM combinational dual lure with the UV LED significantly increased the catch of both OFM and CM in the same trap [85]. The development of smart traps that can differentiate these two species based on moth size and shape may be a useful tool for many pome fruit-growing regions [52].

Dispensers for OFM MD are used over a range from 2.5 to 500 units ha^−1^. However, studies exploring the use of much higher densities of wax drops suggest that this can improve MD. Commercial development and grower adoption was likely slowed due to application costs from specialized equipment and multiple applications [94,169].

### 9.3. European Grape Vine Moth

Monitoring EGVM males with sex pheromone-baited traps has been difficult under MD [170]. We were able to access much fewer studies assessing female mating under MD with EGVM than CM or OFM, likely because much of the work was published in foreign languages in regional journals that were not accessible (some references cited inside [8,171]). All studies with EGVM were conducted using hand-applied dispensers with no data available on the use of aerosol devices (Table 1). This lack of monitoring of female EGVM is likely because a very high proportion of females caught in bait traps are mated both inside and outside MD-treated vineyards [73,170,172,173]. No studies tethering female EGVM to assess MD were found, perhaps because of its small size [171].

The combination of 2-phenylethanol and acetic acid was identified as an attractant for EGVM following extensive work developing dual sex lures for LR species in tree fruits [135,136,137,138,139,143]. The lure has been commercialized by Trécé Inc. (Adair, OK, USA) as an LR lure. Acetic acid and 2-phenylethanol are ubiquitous volatiles released by both microbes and higher plants [174,175]. It is not clear what type of behavioral signal this lure sends to EGVM and leafrollers [140,141]. A second compound found to be attractive for leafrollers, benzyl cyanide, when used with acetic acid, was also attractive for EGVM [141]. However, this compound is not likely to be registered due to human health concerns. These three studies with EGVM did not report the mating status of females or even whether they were being tested under MD.

The first EGVM study with 2-phenylethanol and acetic acid under MD was conducted in Spain and Chile with two MD dispenser types over one and two seasons, respectively [144]. The data demonstrated that the proportion of female mating increased in MD-treated vineyards from the first to the third flights and was lower in a larger and more isolated vineyard. Unfortunately, there were no untreated vineyard comparisons in either country to assess the impact of MD. Hopefully, this lure will be adopted to assess MD in the large-scale management programs ongoing for EGVM in Europe and South America [3,33].

### 9.4. Leafrollers

There are nearly 1000 tortricids considered as pests across many crops, and hundreds of them are referred to as leafrollers [176]. The summaries of 46 studies with the five leafroller species attacking tree fruit in the western United States were collected from 12 papers (Table 1). These included oblique banded leafroller (OBLR), *Choristoneura rosaceana* Harris; the sibling species Pandemis leafroller (PLR), *Pandemis pyrusana* Kearfott; three-lined leafroller (TLLR), *Pandemis limitata* Robinson; fruit tree leafroller (FTLR), *Archips argyrospila* Walker, and orange tortrix (OT), *Argyrotaenia citrana* (Fernald).

The focus of these papers was to compare the disruption achieved with different incomplete and complete blends for these LR species in order to develop a generic blend that is effective for all of them. All of these species use (*Z*)-11-tetradecenyl acetate as a principal pheromone component, and trace amounts of the E-isomer are usually present. The Hamaki-con dispenser with a 93:7 (*Z*,*E*) content was readily available for testing in North America [177]. While OT uses (*Z*)-11-tetradecenyl acetate in combination with (*Z*)-11-tetradecenal, OBLR in eastern North America uses the acetate with (*Z*)-11-tetradecenol and the alcohol plus the aldehyde in the western part of its range [178]. The two Pandemis species use a mixture of acetate with (*Z*)-9-tetradecenyl acetate. All the formulations tested had *Z*:*E* ratios of all four compounds >90:10.

Interestingly, unlike with CM or OFM, the percentage reduction in female mating was significantly lower with tethering than when using VF-baited traps (Table 1). The leafrollers are generally larger moths than the three other tortricids reviewed in this contribution [179,180]. Among LR, OBLR is reported to be the largest tortricid in North America [181]. Thus, VF-baited traps may have remained attractive for a longer period of time with leafroller adults. In comparison, all the tethered females were removed after 1 day. Also, many of the studies used two VFs in the trap. Interception traps were used in one study with OBLR but caught a few moths and were not useful [182].

Data from tethering female OBLR were placed into three groups based on the two-, three-, or four-component blends used in field trials with dispensers. Mean reductions in female mating were very similar among these three groups, 76 to 80%, and not significantly different, F_2,14_ = 0.44, *p* = 0.6545. The generic Hamaki-con rope was widely tested and produced > 90% reductions in mating of tethered females with all four species tested. The extensive research conducted with tethered OBLR and TLLR females using hollow fiber dispensers suggested that a generic blend not including the (*Z*)-9-tetradecenyl acetate could be used for both species [183,184]. One study compared a higher rate of dispensers with an incomplete blend using VF-baited traps and had very high and similar reductions in mean catch as the standard rate [185].

Only one study to date has evaluated LR female mating status (specifically OBLR) under MD using the 2-phenylethanol lure [143]. Nevertheless, OBLR is highly dispersive, and the replicated MD blocks were small and intermingled with untreated blocks. Unfortunately, one large areawide project (150 ha) for OBLR/PLR was conducted without gathering female mating data [99].

## 10. Female Moths and Factors Affecting MD Success

In our review, we are focused on how the proportions of mated females can be so high under MD despite its successful adoption. Stationary assessments suggest that MD is very effective. Researchers all state that to be successful, both a low pest population and a low level of immigration are required. But a lot of data from the active assessments show a relatively high proportion of the females under MD are mated. Looking at stationary and active assessments, respectively, which sample is correct: >80% or <30% disruption of females? If the first estimate is correct, then why does every sample of wild moths dispute this success? Perhaps the methods are biased. If the latter is correct, then what are the factors that can interact to still allow MD to be effective? In this final section of this review, we want to address two points made previously: (*i*) female moths are not just ‘stationary point sources of pheromone’ [186], and (*ii*) the inclusion of factors affecting the mating of female tortricids adds a new dimension in refining the use of MD [13].

### 10.1. Female Movement Within the MD Treatment

Movement of females and patterns of oviposition are important moth behaviors that are difficult to study in the field. Some species move seasonally between different crops, such as OFM between stone and pome fruits [68,187]. Field observations can characterize a static canopy distribution of moths and female calling and resulting patterns of oviposition [59,188]. Tracking moth movement and differentiating the sexes, especially around dusk, is not practical unless non-invasive techniques can be developed [60]. The use of passive interception traps placed in the canopy was shown to be unbiased for female CM mating status and has a sex ratio favoring males [64]. Similarly, the mating status of females with light traps provides an accurate estimate of mating [189]. Whether the mating status of CM females caught with the four-component dual sex lure is biased has not yet been determined [50]. The proportions of mated CM caught with this dual sex lure across different MD-treated orchards have varied from 0.20 to 0.65 [96]. The addition of a UV LED increased catch but did not alter the proportion of females mated [84].

Female moth flight can be prompted to acquire food and water resources. Research is mixed on whether tortricid females require water and sugary resources to reach their full fecundity—[190,191] vs. [192,193]. These are all laboratory studies where the resources are provided at the start of the test. Female CM and OBLR that are not allowed to mate for several days do have a significantly lower fecundity if they cannot have access to honey water (A.L.K., unpublished data). However, this was not found with EGVM [194]. Field observations of tortricid adults feeding are more limited [195,196]. Natural sugary resources from flowers and dropped fruit may occur inside or outside MD orchards. Interestingly, both acetic acid and 2-phenylethanol are major constituents of both sugary baits and several dual sex lures [70,197]. Unexpectedly, the great variability in female OFM mating status reported from bait traps (8–94%) suggests these data can be a useful measure, and most likely, they should be used in the center of large blocks to minimize the catch of immigrant moths [74,77].

Host olfactory cues mediate oviposition, and tortricid species lay either individual or clusters of eggs. Interspecific egg avoidance contributes to local and dispersive mated female movement among hosts but probably does not impact virgin females [198,199].

Larval aggregation pheromone for CM has been suggested as an adaptive behavior to procure mates that may work against MD [200]. Larvae aggregate to overwinter and pupate in the spring on tree trunks [201]. Males emerge before females on average, and sex pheromone release begins in the female pupal stage. The hypothesis is that males remain near the female pupae, and this increases the mating probability and makes MD less effective. However, there are no observational data confirming that this occurs, and CM female mating on tree trunks near overwintering sites has not been observed. In comparison, OFM larvae have a similar overwintering strategy as CM, but there is no evidence of a larval aggregation sex pheromone [202]. This difference in larval behaviors of the two tortricid species correlates with the difficulty in managing CM vs. OFM with MD. While this species difference is not apparent with the stationary assessment methods of female mating, in general, male catch in sex pheromone-baited traps is much easier to shut down with OFM than CM [203,204].

### 10.2. Immigration into MD Treatment

Female moths moving deliberately or accidentally into and out of host crops is to be expected. Clearly, MD treatment being adjacent to an infested host tree or abandoned vineyard is going to be a management problem. Flight mills have been used in the laboratory to compare sexes and the influence of mating status and age of tortricids on flight distance or duration [205,206,207]. While immigration of females across landscapes is a key factor influencing orchard management success, it is not well studied among MD-treated hosts [68,208,209].

Perhaps the sex ratio of moths caught with the various dual sex lures may be useful to view the importance of immigration. For example, traps baited with the quaternary lure developed for CM can have very even sex ratios under MD [129,130]. Orchards where the proportion of males is elevated may have a greater occurrence of immigrants than local moths, but the female mating status among immigrants has not been determined. Measuring the sex ratio of moths and female mating status in traps placed on the perimeter vs. in an internal grid could perhaps show this disparate movement between the sexes and the influence of immigration [210,211]. In addition, measuring the level of pest pressure gradient within the MD-treated blocks and in the surrounding landscape may help to define ‘hot spots’ areas and high-risk edges where to implement the MD dispensers’ density.

### 10.3. Pheromone Autodetection and Female Moth Movement

Various studies have found that female tortricids can detect their own sex pheromone [212,213,214,215,216]. Different impacts on female behavior from their pre-exposure to sex pheromone have been documented, including temporal shifts and duration in female calling and flight [62,217]. However, multiple studies with OFM have had mixed results [214,218], and no effect was found for EGVM [219]. Another study with OFM failed to identify any sex pheromone-specific olfactory receptor neurons on female antennae and suggested that autodetection in unlikely in this species [220].

Two hypotheses on the impact of autodetection on female movement and spacing have been proposed (summarized in [62,221]). First, because females can detect their sex pheromone, they sense in MD blocks that they are surrounded by females competing for mates. The early work by Pearson et al. [186] with a day flying sesiid, *Vitacea polistiformis* Harris, demonstrated that females’ behaviors are impacted by MD, including increased intra-canopy movement and activity levels before (−) and after (+) bouts of calling. Their model suggested that if female movement patterns avoided overlapping sex pheromone plumes, then their mating success increased. A second study with EGVM found that virgin females engaged in more short flights but called less frequently in MD-treated vineyards [13]. The conflict between virgin females moving to more sites but calling less in MD-treated vineyards may cancel out any effect on net oviposition. Whether unmated females would disperse out of MD-treated hosts in search of mates seems unlikely, especially with tortricid males mating frequently. It is questionable whether enhanced fecundity gained from selective mating with a virgin male would outweigh the accumulating cost of signaling over time [222,223].

A second hypothesis has been that mated females might want to leave MD-treated hosts to avoid future larval resource competition. This idea is also probably not relevant for these tortricids as the available larval resources are generally great, especially for the shoot and foliage feeders. While CM will disperse from non-bearing orchards, these barren hosts are unlikely to have had MD applied [224].

Considering the advances in the volatile organic compounds collection techniques, actual field data on the dose and fragmentation of synthetic pheromone compounds in the atmosphere of MD-treated orchards and vineyards could be obtained. This information could help understand better the concentration of synthetic pheromones in the air and its effect on both MD success and pheromone autodetection of wild females.

### 10.4. Multiple Mating

All of these tortricids can mate during their first 24 h and will begin to lay eggs the following day (see respective chapters in Van der Geest and Evenhuis [1]). Also, both sexes can mate more than once and females, not males, have a refractory period between subsequent matings. Some studies have found that multiple-mated females can have a higher fecundity [146]. Several MD studies with CM found that the reduction in multiple-mated females was much greater than singly mated [96,145,146,147]. The occurrence of males mating more than once is inferred from the presence of smaller spermatophores [146]. Multiple-mated females tend to have at least one small spermatophore [225]. A higher proportion of mated females under MD had smaller spermatophores, suggesting that they were mated by an experienced older male [146]. The concept of a ‘super male’ that can successfully mate under MD was proposed in this paper for males that are better able to discriminate (‘learn’) the female’s subtle natural blend among an overflooding background of synthetic chemicals. Females tend to mate only once per night, so females that are multiple-mated are, on average, older. The significant reductions in multiple-mated CM females under MD could suggest that there was a delay in mating. Unfortunately, most researchers have not reported the number of spermatophores possessed by mated females in MD-treated orchards.

### 10.5. Delay in Mating

Measuring the proportion of mated females under MD only provides information on one mechanism of its impact on pest populations [92]. The realized female fecundity and female density under MD are the two most important measures to assess MD. Several factors can influence fecundity, including the age of the moths when they mate [226]. Delayed mating is typically associated with lower fecundity and fertility and extended longevity under laboratory conditions [227,228,229,230]. Demonstrating the occurrence of MD causing a delay in female mating among tortricids has only been reported from one field study of CM [231]. In this study, it took three days for the proportions of mated females observed on day 1 with no MD (ca. 40%) to occur in an orchard treated with Isomate dispensers at 1000 ha^−1^. No data were collected on oviposition in the field.

Tortricids can begin to mate within 24 h post-eclosion, and mating generally peaks at 2–3 days (see species’ chapters in Van der Geest and Evenhuis [1]. The impact of a 2- to 3-day delay in female mating on fecundity observed in the laboratory has been variable. For example, CM had 10, 21, or 29% reductions in fecundity in three studies [227,230,231]. A 2-day delay reduced fecundity 47% and 54% with PLR and OBLR, respectively [230,232]. Studies with either a 3-day delay with EGVM or a 2-day with OFM found fecundity was reduced by 6% and 13%, respectively [228,229]. These variable results could be due to differences in laboratory colonies or experimental laboratory conditions, such as relative humidity. These studies all reported a much higher reduction in fecundity when the delay in mating was much longer, 62–87% with ≥6 d. Also, there was a greater reduction when both sexes were aged [232]. However, the longevity of female tortricids in managed orchards and vineyards has not been well studied under natural conditions [233]. Therefore, it remains unclear how important or how often a delay in mating occurs with any MD tactic [228].

Another population factor related to a mating delay would be that female mortality would be higher before oviposition begins or peaks [226]. Adoption of MD is often associated with reduced spray practices, and thus, less disruption of natural controls would occur in tree fruit and grapes [5,8,195,234]. Natural adult mortality from a suite of general predators such as spiders or bats and birds could be important [171,235,236,237]. The transition in spray programs from broad-spectrum to selective materials that co-occurred with rapid MD adoption, however, may have reduced this impact from natural enemies [238]. Adult exposure to insecticide residues is also important, and even the more selective insecticides can have sublethal effects that impact fecundity [239,240,241].

Another factor to consider is the interaction of delayed mating with intra-orchard movement. If unmated females are more likely to move as they age to find a mate, this behavior will increase the proportion of mating, but these older mated females will still have a lower fecundity. This behavior has been suggested by us as to why the female disruption measured by active and stationary assessments may differ so widely.

## 11. Take-Home Messages

This contribution provides an overview of the various ways that the mating success of female tortricids can impact behavior-modifying technologies focused on several key pests of tree fruit and grapes. Then, a focus on the literature of female mating under MD applications is given. Finally, the authors address how additional female behavioral factors under MD may interact to suppress pest populations and levels of crop injury. Ten major points are evident from this review:

1. MD has been widely adopted for the tortricid species considered in this contribution.
2. Current MD programs are not highly effective in preventing female mating.
3. The success of MD may be a combination of reduced mating, a delay in mating, changes in female behaviors, and enhanced natural control. These factors are not well documented in the field.
4. The success of MD can be compromised if mated females enter MD-treated plots; thus, exclusion methods, such as netting or areawide expansion of MD, provide the best crop protection.
5. The concept that the complete sex pheromone blend provides the greatest disruption has not been adopted.
6. There is little evidence that moth density impacts mating success under MD.
7. Widely used stationary assessment methods such as tethering or virgin female-baited traps are not reflective of the mating status of wild female populations.
8. Current dispenser/sprayable rates of MD are not likely optimal and could be implemented considering the female moths’ mating status.
9. New dual sex lures are convenient and can be used to assess the phenology and seasonal densities of female populations.
10. New dual sex lures can be used to evaluate the success of current MD programs and guide the development of more effective programs.

In conclusion, increased knowledge on how these factors (i.e., mating disruption, delay of mating, immigration, and changes in female behaviors) influence the success of MD can allow us to develop more effective management programs for these key pest species.

## Figures and Tables

**Table 1:** Summary of the various assessment methods, including tethered females, virgin female-baited traps, interception traps, bait traps, light traps, and traps with various attractant lures used to measure the reduction in female mating due to the use of MD technologies for CM, OFM, EGVM, and LR.

Species	Assessment	Number of Studies	Mean ± SE Percent Reduction in Matings Compared with Untreated Sites ^a^
CM	Tethered VF	11	82.8 ± 4.1 A
CM	VF-baited trap	22	75.5 ± 4.4 A
CM	Interception traps	7	25.7 ± 7.3 B
CM	Lure-baited traps	24	11.0 ± 1.6 B
Kruskal–Wallis			*W* = 47.2, *p* < 0.0001
OFM	Tethered VF	4	92.0 ± 5.9 A
OFM	VF-baited trap	8	94.3 ± 1.8 A
OFM	Bait bucket	7	16.9 ± 11.0 B
OFM	Lure-baited trap	4	5.3 ± 3.1 B
Kruskal–Wallis			*W* = 16.4, *p* < 0.0001
EGVM	VF-baited trap	5	98.2 ± 1.1 A
EGVM	Bait bucket	4	2.8 ± 1.5 B
EGVM	PET + AA	2 ^b^	-
Kruskal–Wallis			*W* = 6.2, *p* = 0.0127
LR	Tethered VF	33	81.8 ± 2.5 B
LR	VF-baited trap	10	94.0 ± 2.2 A
LR	Bait bucket	1 ^b^	-
LR	PET + AA	2 ^b^	5.0 ± 0.0
ANOVA			*F*_1,41_ = 8.19, *p* = 0.0066

Per each species, mean values followed by a different letter are significantly different (*p* < 0.05). ^a^ Data are the reduction in the proportion of tethered females mated, VF-baited traps catching ≥ 1 male, the mean male catch per trap, or of mated wild females caught on interception traps or traps with baits or lures. ^b^ Data not included in the analysis due to the limited number of replicates.

## Data Availability

Raw data summarized from the review of the literature and used for the analyses is available in the public repository at https://doi.org/10.5281/zenodo.14752202.

## Appendix A

**Table A1:** Research trials including assessments of female mating status under different mating disruption programs for codling moth (CM), *Cydia pomonella* L. (Lepidoptera: Tortricidae). MD = data from mating disruption plots; UTC = data from untreated check plots without mating disruption (control).

Species-Trial #	Assessment ^a^	MD Dispensing System, Active Ingredient, Rate per Hectare	Percentage ± SE Females Mated or VF Traps (%) Catching or Males (*Mean*) Caught per VF Trap		Reduction (%) in Mated Females with MD vs. UTC	Reference
			UTC	MD		
CM-1	T	Fibers, PH, 250 per tree	44	0	100	[15]
CM-2	T	Isomate, PH, 1000	40	4	90	[59]
CM-3	T	Isomate, PH, 1000	27	3.5	87	[153]
CM-4	T	Cidetrak, PH/PE, 1000	27	1.9	93	[153]
CM-5	T	Fibers, PH, 90,000	48	17.5 ± 3.2	64	[242]
CM-6	T	Fibers, PH + Isomate, PH, 1000	25	9.3 ± 1.5	63	[242]
CM-7	T	Isomate, PH, 1000	46	18.3 ± 1.8	60	[243]
CM-8	T	Isomate, PH, 1000	35	4.8 ± 2.3	86	[154]
CM-9	T	Cidetrak, PH/PE, 1000	32	2.5 ± 0.5	92	[154]
CM-10	T	Isomate, PH, 1000	38	4	89	[159]
CM-11	T	NoMate, PH, 600–800	38	5	87	[159]
CM-12	VF-10	Experimental, codlemone acetate	-	-	100	[151]
CM-13	VF-10	Experimental, 4 PH isomers	-	-	86.8	[161]
CM-14	VF-10	Experimental, codlemone	-	-	68.7	[161]
CM-15	VF-1/2	Isomate, PH, 1000	16.1 (*mean*)	0	100	[164]
CM-16	VF-1/2	Isomate, PH, 2000	3.1 (*mean*)	0	100	[164]
CM-17	VF-2	Isomate, PH, 1000	48.7 ± 17.5	3.7 ± 2.1	92	[160]
CM-18	VF-2	Isomate, PH, 1000	88	5.5 ± 1.8	94	[160]
CM-19	VF-2	Fibers, PH-1 comp, 5000–100,000	74	15.7 ± 2.6	79	[160]
CM-20	VF-2	Fibers, PH-3 comp, 5000–100,000	74	19.0 ± 5.5	74	[160]
CM-21	VF-3/4	Aerosol, PH, 2.3 units	10.1 (*mean*)	0.2	98	[244]
CM-22	VF-2	MEC, PH airblast, 250 mL	30	13	57	[158]
CM-23	VF-2	MEC, PH Low volume, 250 mL	30	3	90	[158]
CM-24	VF-2	Checkmate, PH, 500	35	3	91	[155]
CM-25	VF-2	Cidetrak, PH, 1000	35	7	80	[155]
CM-26	VF-2	Cidetrak, PH/PE, 500	35	4	89	[155]
CM-27	VF-2	Cidetrak, PH/PE, 1000	35	3.5	90	[155]
CM-28	VF-2	Isomate, PH, 1000	35.5 ± 7.5	18.0 ± 1.0	49	[156]
CM-29	VF-2	Cidetrak, PH, 1000	35.5 ± 7.5	14.5 ± 2.5	59	[156]
CM-30	VF-2	Cidetrak, PH/PE, 1000	35.5 ± 7.5	9.2 ± 0.7	74	[156]
CM-31	VF-2	Isomate, PH, 1000	28.0 ± 5.0	15.5 ± 0.5	45	[156]
CM-32	VF-2	Cidetrak, PH, 1000	28.0 ± 5.0	13.0 ± 6.0	54	[156]
CM-33	VF-2	Cidetrak, PH/PE, 1000	28.0 ± 5.0	9.7 ± 2.1	65	[156]
CM-34	VF-2	Cidetrak, PH, 800	3.2 ± 2.9 (*mean*)	2.4 ± 0.9	25	[61]
CM-35	VF-2	Cidetrak, PH/PE, 800	3.2 ± 2.9 (*mean*)	1.1 ± 0.4	66	[61]
CM-36	VF-2	Cidetrak, PH/PE, 80	3.2 ± 2.9 (*mean*)	0.4 ± 0.4	87	[61]
CM-37	VF-2	Isomate, PH, 800	3.2 ± 2.9 (*mean*)	0.8	75	[61]
CM-38	PI	Isomate, PH, 1000	-	62	-	[59]
CM-39	PI	Isomate, PH, 1000	85	40	53	[231]
CM-40	PI	Isomate, PH, 1000	88	60	32	[64]
CM-41	PI	Isomate, PH, 1000	-	59.0 ± 15.3	-	[64]
CM-42	PI	Isomate, PH, 1000	80	77	4	[11]
CM-43	PI	Cidetrak, PH, 800	49 ± 6.0	66.5 ± 7.6	0	[61]
CM-44	PI	Cidetrak, PH/PE, 800	49 ± 6.0	39 ± 1.5	20	[61]
CM-45	PI	Cidetrak, PH/PE, 80	49 ± 6.0	28.5 ± 1.5	42	[61]
CM-46	PI	Isomate, PH, 800	49 ± 6.0	35	29	[61]
CM-47	LT	Isomate, PH, 1000	-	37	-	[189]
CM-48	LT	Isomate, PH, 1000	-	84 ± 1.0	-	[11]
CM-49	PE	Isomate, PH, 1000	-	93 ± 2.0	-	[11]
CM-50	PE	Isomate/Cidetrak, PH, 1000	81.0 ± 1.7	71.9 ± 7.4	10	[145]
CM-51	PE	Cidetrak/Isomate, PH/PE, 1000	81.0 ± 1.7	84.6 ± 15.4	0	[145]
CM-52	PE	Sprayable, PH, 250 mL	78.4 ± 3.3	81.1 ± 6.1	0	[145]
CM-53	PE	Sprayable, PH/PE, 250 mL	84.3 ± 0.2	80.0 ± 5.2	5	[145]
CM-50B ^b^	PE	Isomate/Cidetrak, PH, 1000	2.9	2.2 ± 0.4	24	[145]
CM-51B	PE	Cidetrak/Isomate, PH/PE, 1000	2.9	0	100	[145]
CM-52B	PE	Sprayable, PH, 250 mL	9.7 ± 0.8	4.9 ± 0.7	49	[145]
CM-53B	PE	Sprayable, PH/PE, 250 mL	6.1 ± 0.5	2.7 ± 1.2	56	[145]
CM-54	PE	Isomate, PH, 1000	84 ± 2.0	85 ± 2.0	0	[11]
CM-55	PE	Isomate, PH, 500–750	89	84.5	5	[146]
CM-56	PE	Isomate, PH, 1000	89	81	9	[146]
CM-57	PE	Isomate, PH, 1000	89	77.5	13	[146]
CM-55B ^b^	PE	Isomate, PH, 500–750	18.5 ± 13.5	3.5 ± 2.5	81	[146]
CM-56B	PE	Isomate, PH, 1000	18.5 ± 13.5	3.0 ± 2.0	84	[146]
CM-57B	PE	Isomate, PH, 1000	18.5 ± 13.5	3.5 ± 1.5	81	[146]
CM-58	PE, PH/PE	Isomate, PH, 1000	-	40	-	[245]
CM-59	PH/PE + AA	Cidetrak, PH/PE, 800	-	55	-	[246]
CM-60	PH/PE + AA	Cidetrak Meso, PH/PE, 80	-	60	-	[246]
CM-61	PH/PE + AA	Cidetrak Meso, PH, 80	-	36	-	[246]
CM-62	PH/PE	DAMEC, PE, 5 g a.i.	72	70	3	[157]
CM-63	PH/PE	PHMEC, PH, 25 g a.i.	72	58	19	[157]
CM-64	PH/PE	PHMEC + DAMEC, PH/PE, 25 + 5 g	72	64	11	[157]
CM-65	PH/PE	DAMEC, PE, 5 g a.i.	74	65	12	[157]
CM-66	PH/PE	PHMEC, PH, 25 g a.i.	74	58	26	[157]
CM-67	PH/PE	PHMEC + DAMEC, PH/PE, 25 + 5 g	74	64	14	[157]
CM-68	PH/PE	Meso, PH/PE, 50	87 ± 3.0	69 ± 7.2	21	[147]
CM-69	PH/PE	Meso, PH, 50	84 ± 1.0	73.5 ± 0.5	12	[147]
CM-70	PH/PE	Isomate Ring, PH, 50, 170	84.5 ± 0.5	82 ± 3.0	3	[147]
CM-68-B ^b^	PH/PE	Meso, PH/PE, 50	22.8 ± 2.7	5.8 ± 0.6	75	[147]
CM-69-B	PH/PE	Meso, PH, 50	23 ± 6.0	13 ± 0.0	43	[147]
CM-70-B	PH/PE	Isomate Ring, PH, 50, 170	27 ± 2.0	18.5 ± 1.5	31	[147]
CM-71	4K	Several PH and PH/PE	82 ± 4.1	68 ± 5.2	17	[97]
CM-72	4K	Cidetrak, PH/PE	85 + 3.4	65 ± 3.0	24	[97]
CM-73	4K	Several PH dispensers	-	90 ± 3.0	-	[97]
CM-74	4K	Cidetrak, PH/PE, 1000	76 ± 2.0	60 ± 4.0	21	[96]
CM-75	4K	Cidetrak, PH/PE, 500	83 ± 2.0	70 ± 5.0	16	[96]
CM-74-B ^b^	4K	Cidetrak, PH/PE, 1000	25	6.5	74	[96]
CM-75-B	4K	Cidetrak, PH/PE, 500	29.5	5	83	[96]
CM-76	4K	Cidetrak, PH/PE, 800	-	72.8 ± 0.5	-	[84]
CM-77	4K + light	Cidetrak, PH/PE, 800	-	71.6 ± 3.4	-	[84]
CM-78	4K	Cidetrak Meso, PH, 80	83 ± 3.9	78	6	[107]
CM-79	4K	Isomate, PH, 1000	83 ± 3.9	87	0	[107]
CM-80	4K	Cidetrak, PH/PE, 500	83 ± 3.9	70	16	[107]
CM-81	4K	Cidetrak, PH/PE, 800	81 ± 7.0	65	20	[85]
CM-82	4K + light	Cidetrak, PH/PE, 800	62 ± 9.0	50	19	[85]

^a^ Assessment methods include tethered virgin females (T), virgin female-baited traps and the number of moths used per trap (VF-#), passive interception traps (PI), light traps (LT), and lures: PHEROCON^®^ CMDA with pear ester (PE), PHEROCON^®^ CMDA Combo with pear ester in combination with sex pheromone (PH/PE) and plus acetic acid co-lure (AA), and PHEROCON^®^ Megalure CM Dual 4K (4K) with or without a UV LED (light). ^b^ Gray-shaded rows (CM-XX-B’s) are for the occurrence of multiple-mated female codling moth.

**Table A2:** Research trials including assessments of female mating status under different mating disruption programs for Oriental fruit moth (OFM), *Grapholita molesta* Busck (Lepidoptera: Tortricidae). MD = data from mating disruption plots; UTC = data from untreated check plots without mating disruption (control).

Species-Trial #	Assessment ^a^	MD Dispensing System, Rate per Hectare	Percentage ± SE Females Mated or VF Traps (%) Catching or Males (*Mean*) Caught per VF Trap		Reduction (%) in Mated Females with MD	Reference
			UTC	MD		
OFM-83	T	Splat drops, 3–10 per tree	57	14.5 ± 5.5	75	[169]
OFM-84	T	Splat drops, 30–100 per tree	57	0.0	100	[169]
OFM-85	T	Splat drops, 10 mL per tree	39.4 ± 8.8	2.7 ± 1.3	93	[94]
OFM-86	T	Splat drops, 8 mL per tree	27	0	100	[247]
OFM-87	VF-3	Fibers, 1200–68,000	139 (*mean*)	3.7 ± 2.2	97	[22]
OFM-88	VF-3	Fibers, 700–61,000	139 (*mean*)	1.0 ± 1.0	99	[22]
OFM-89	VF-1	Isomate M, 1000–1300	39 (*mean*)	0	100	[168]
OFM-90	VF-1	Confuse drops, 520–580	2.0 (*mean*)	0.15	92	[248]
OFM-91	VF-1	Confuse drops, 520–580	6.0 (*mean*)	0.5	92	[248]
OFM-92	VF-1	Confuse drops, 520–580	10.0 (*mean*)	0.2	98	[248]
OFM-93	VF-1	Confuse OFM, 570	5.0 (*mean*)	0.5 ± 0.2	90	[249]
OFM-94	VF-1	Paraffin, 35–247	0.95 (*mean*)	0.13 ± 0.06	86	[249]
OFM-95	BA	Microcaps, 0.5–2 per tree	100	94	6	[74]
OFM-96	BA	Microcaps, 3–4 per tree	95.5 ± 1.5	23	76	[75]
OFM-97	BA	Isomate, 1000	97.5 ± 0.9	62.3 ± 10.7	36	[167]
OFM-98	BA	Isomate-M, 240–900	-	78.6	-	[195]
OFM-99	BA	Isomate Rosso, 480	92.3	92.8	0	[195]
OFM-100	BA	Isomate M100, 250	-	75 ± 12.0	-	[69]
OFM-101	BA	Confuse OFM, 580	70 ± 8.0	73	0	[250]
OFM-102	BA	Wax dollops, 590	70 ± 8.0	83.5 ± 16.5	0	[250]
OFM-103	BA	Isomate M-100, 250	70 ± 8.0	74.5 ± 25.5	0	[250]
OFM-104	BA	Checkmate OFM SL+, 625	-	46	-	[71]
OFM-105	BA-AJAR	Checkmate OFM SL+, 625	-	27.5 ± 2.5	-	[71]
OFM-106	BA	Rak 5, 600	-	34	-	[76]
OFM-107	BA-AJAR	Isomate Rosso, 600	-	51.0 ± 5.0	-	[76]
OFM-108	BA	Splat Grafo, 1 kg	-	7.5 ± 2.3	-	[77]
OFM-109	BA-AJAR	Splat Grafo, 1 kg	-	18.7 ± 4.7	-	[77]
OFM-110	OCD	Cidetrak OFM-L, 450	-	38	-	[132]
OFM-111	OCD	Isomate M100, 400	-	20.5 ± 3.5	-	[132]
OFM-112	OCD	No MD	71.5 ± 1.3	-	-	[84]
OFM-113	OCD	Cidetrak OFM L, 500	67	91	0	[85]
OFM-114	OCD	Isomate OFM/PTB TT, 500	77	68	12	[85]
OFM-115	OCD	Semios OFM Aerosol, 2.5	93	85	9	[85]
OFM-116	OCD + light	Two dispensers, 250–500	74.0 ± 3.1	83.0 ± 4.2	0	[85]
OFM-117	OCD	Checkmate OFM puffer, 2.5	-	69	-	[85]
OFM-118	OCD + light	Checkmate OFM puffer, 2.5	-	72	-	[85]

^a^ Assessment methods include tethered virgin females (T), virgin female-baited traps and the number of moths used per trap (VF-#), bait traps (BA), AJAR bait traps (BA-AJAR), and the PHEROCON^®^ OFM Combo Dual (OCD) lure with or without a UV LED (light).

**Table A3:** Research trials including assessments of female mating status under different MD programs for European grape vine moth (EGVM), *Lobesia botrana* Denis & Schiffermüller (Lepidoptera: Tortricidae). MD = data from mating disruption plots; UTC = data from untreated check plots without mating disruption (control).

Species-Trial #	Assessment ^a^	MD Dispensing System, Rate per Hectare	Percentage ± SE Females Mated or VF Traps (%) Catching or Males (*Mean*) Caught per VF Trap		Reduction (%) in Mated Females with MD	Reference
			UTC	MD		
EGVM-119	VF-1	BASF, 2000	128 (*mean*)	6	95	[251]
EGVM-120	VF-5	Ecodian LB, 1600	7.5 ± 4.7 (*mean*)	0.0	100	[252]
EGVM-121	VF-5	Isonet L, 500	7.5 ± 4.7 (*mean*)	0.3	96	[252]
EGVM-122	VF-5	Ecodian LB, 1600	4.3 ± 3.6 (*mean*)	0	100	[252]
EGVM-123	VF-5	Isonet L, 500	4.3 ± 3.6 (*mean*)	0	100	[252]
EGVM-124	BA	Unspecified, 400	98	95	3	[73]
EGVM-125	BA	Rak 2, 600	86	87	0	[172]
EGVM-126	BA	Rak 2, 400	83	82	1	[172]
EGVM-127	BA	Rak 2, 400	91	85	7	[172]
EGVM-128	PET + light	Lobetec, 400	-	NA–25–78	-	[144]
EGVM-129	PET + light	Isonet L, 500	-	0–35–26		[144]
EGVM-130	PET + light	Isonet L, 500	-	48–70–83	-	[144]

^a^ Assessment methods include virgin female-baited traps and the number of moths used per trap (VF-#), bait traps (BA), and the 2-phenylethanol plus acetic acid (PET) lure with or without a LED (light). Data from trials #128–130 were a combined data set with or without either a UV, blue, or green LED and are shown separately for three seasonal EGVM flights (NA—data were not collected for the first flight).

**Table A4:** Research trials including assessments of female mating status under different MD programs for five leafroller (LR) species: oblique banded leafroller (OBLR), *Choristoneura rosaceana* Harris; Pandemis leafroller (PLR), *Pandemis pyrusana* Kearfott; three-lined leafroller (TLLR), *Pandemis limitata* Robinson; fruit tree leafroller (FTLR), *Archips argyrospila* Walker; and orange tortrix (OT), *Argyrotaenia citrana* Fernald (Lepidoptera: Tortricidae). MD = data from mating disruption plots; UTC = data from untreated check plots without mating disruption (control).

Species-Trial #	Assessment ^a^	MD Dispensing System, Active Ingredient, Rate per Hectare	Percentage ± SE Females Mated or VF Traps (%) Catching or Males (*Mean*) Caught per VF Trap		Reduction (%) in Mated Females with MD	Reference
			UTC	MD		
LR-131-OT	T	Hamaki-con, 2-comp, 1000	35	2	95	[253]
LR-132-OT	T	Isomate OT, 3-comp, 1000	35	0	100	[253]
LR-133-OBLR	T	Fibers, 2-comp, 1000	40	3	92	[254]
LR-134-OBLR	T	Fibers, 3-comp, 1000	40	7	82	[254]
LR-135-OBLR	T	Fibers, 4-comp, 1000	40	5	87	[254]
LR-136-OBLR	T	Fibers, 4-comp, 1000	-	-	80	[254]
LR-137-OBLR	T	Fibers, 4-comp, 1000	70	17	76	[254]
LR-138-OBLR	T	Fibers, 2-comp, 1000	70	17	76	[254]
LR-139-OBLR	T	Fibers, 4-comp, 250	52	18	65	[254]
LR-140-OBLR	T	Fibers, 2-comp, 250	52	15	71	[254]
LR-141-OBLR	T	Isomate, incomplete blends, 990	41.0 ± 5.0	6.5 ± 6.5	84	[182]
LR-142-OBLR	T	Membrane, complete blend, 990	13.5 ± 7.5	2.0 ± 2.0	85	[182]
LR-143-OBLR	T	Fibers, 2-comp, 1000	65	10.0	85	[183]
LR-144-OBLR	T	Fibers, 4-comp + TLLR, 1000	50	17	66	[183]
LR-145-OBLR	T	Fibers, 2–3-comp + TLLR, 1000	45	6.5 ± 3.5	86	[183]
LR-146-OBLR	T	Isomate CM/LR, incomplete, 500	25.9	7.8	70	[255]
LR-147-OBLR	T	3 dispensers, 2 comp, 1000	56.3 ± 7.3	13.3 ± 3.4	76	[256]
LR-148-OBLR	T	3 dispensers, 3 comp, 1000	56.3 ± 7.3	14.8 ± 2.2	74	[256]
LR-149-OBLR	T	Membrane, 4 comp, 1000	66	24	64	[256]
LR-150-OBLR	T	Hamaki-con, incomplete, 1000	20.8	4.9	76	[256]
LR-151-OBLR	T	Hamaki-con, incomplete, 1000	4.6	0.3	94	[256]
LR-152-TLLR	T	Fibers, OBLR complete, 1000	58	10	83	[183]
LR-153-TLLR	T	Fibers, OBLR 2–3 comp, 1000	55.0 ± 3.0	14.0 ± 6.0	75	[183]
LR-154-TLLR	T	Fibers, TLLR complete, 1000	53	39	26	[183]
LR-155-TLLR	T	Fibers, TLLR/OBLR complete	80	3	96	[183]
LR-156-TLLR	T	Fibers, TLLR complete + OBLR	80	0	100	[183]
LR-157-TLLR	T	Isomate CM/LR, incomplete, 500	32.6	5.6	83	[255]
LR-158-PLR	T	Hamaki-con, incomplete, 1000	69	8	88	[257]
LR-159-PLR	T	Membrane, complete, 1000	69	0	100	[257]
LR-160-PLR	T	Membrane, incomplete, 1000	69	8	88	[257]
LR-161-PLR	T	Membrane, incomplete, 1000	69	10	86	[257]
LR-162-PLR	T	Hamaki-con, incomplete, 1000	37	3	92	[257]
LR-163-PLR	T	Membrane, complete, 1000	37	1	97	[257]
LR-164-OT	VF-2	Hamaki-con, incomplete, 1000	76	8	96	[253]
LR-165-OT	VF-2	Isomate OT, complete, 1000	76	0	100	[253]
LR-166-FTLR	VF-2	Hamaki-con, incomplete, 1000	153.5 (*mean*)	1.0	99.3	[185]
LR-167-FTLR	VF-2	Hamaki-con, incomplete, 2000	153.5 (*mean*)	0.3	99.8	[185]
LR-168-OBLR	VF-1	Fibers, complete 1000	50	3.0 ± 2.0	94	[184]
LR-169-OBLR	VF-2	Rope, 2 comp, 1000	74 ± 12	8	89	[256]
LR-170-OBLR	VF-2	Rope, 3 comp, 1000	74 ± 12	16	78	[256]
LR-171-OBLR	VF-2	Hamaki-con, incomplete, 1000	36.2	0.4	99	[256]
LR-172-OBLR	VF-2	Hamaki-con, incomplete, 1000	43.8	1.3	97	[256]
LR-173-OBLR	VF-2	3M MEC-LR, incomplete, 100 g	50 (*mean*)	10 (*mean*)	80	[258]
LR-174-TLLR	VF-1	Fibers, incomplete, 1000	62	7.5 ± 2.5	88	[184]
LR-175-OBLR	BA	Full blend, 494–988	-	69.3 ± 3.1	-	[88]
LR-176-OBLR	PET	Aerosol, 2.5	82	78	5	[143]
LR-177-OBLR	PET	CM/LR Meso, 80	95	90	5	[143]

^a^ Assessment methods include tethered virgin females (T), virgin female-baited traps and the number of moths used per trap (VF-#), bait traps (BA), and the 2-phenylethanol plus acetic acid (PET) lure.
